# UVB phototherapy in an outpatient setting or at home: a pragmatic randomised single-blind trial designed to settle the discussion. The PLUTO study

**DOI:** 10.1186/1471-2288-6-39

**Published:** 2006-08-01

**Authors:** Mayke BG Koek, Erik Buskens, Paul HA Steegmans, Huib van Weelden, Carla AFM Bruijnzeel-Koomen, Vigfús Sigurdsson

**Affiliations:** 1 Department of Dermatology/Allergology (G02.124), University Medical Center Utrecht, Heidelberglaan 100, 3584 CX Utrecht, The Netherlands; 2 Julius Center for Health Sciences and Primary Care (Str. 6.131), University Medical Center Utrecht, Heidelberglaan 100, 3584 CX Utrecht, The Netherlands; 3 Department of Dermatology, St. Antonius Hospital, Koekoekslaan 1, 3435 CM, Nieuwegein, The Netherlands

## Abstract

**Background:**

Home ultraviolet B (UVB) treatment is a much-debated treatment, especially with regard to effectiveness, safety and side effects. However, it is increasingly being prescribed, especially in the Netherlands. Despite ongoing discussions, no randomised research has been performed, and only two studies actually compare two groups of patients. Thus, firm evidence to support or discourage the use of home UVB phototherapy has not yet been obtained. This is the goal of the present study, the PLUTO study (Dutch acronym for "national trial on home UVB phototherapy for psoriasis").

**Methods:**

We designed a pragmatic randomised single-blind multi-centre trial. This trial is designed to evaluate the impact of home UVB treatment versus UVB phototherapy in a hospital outpatient clinic as to effectiveness, quality of life and cost-effectiveness. In total 196 patients with psoriasis who were clinically eligible for UVB phototherapy were included. Normally 85% of the patients treated with UVB show a relevant clinical response. With a power of 80% and a 0.05 significance level it will be possible to detect a reduction in effectiveness of 15%. Effectiveness will be determined by calculating differences in the Psoriasis Area and Severity Index (PASI) and the Self Administered PASI (SAPASI) scores. Quality of life is measured using several validated generic questionnaires and a disease-specific questionnaire. Other outcome measures include costs, side effects, dosimetry, concomitant use of medication and patient satisfaction. Patients are followed throughout the therapy and for 12 months thereafter. The study is no longer recruiting patients, and is expected to report in 2006.

**Discussion:**

In the field of home UVB phototherapy this trial is the first randomised parallel group study. As such, this trial addresses the weaknesses encountered in previous studies. The pragmatic design ensures that the results can be well generalised to the target population. Because, in addition to effectiveness, aspects such as quality of life and cost-effectiveness are also taken into consideration, this study will produce valuable evidence to either support or discourage prescription of home UVB phototherapy.

**Trial registration:**

Current controlled trials/Nederlands Trial register: ISRCTN83025173. Clinicaltrials.gov: NCT00150930

## Background

About 2% to 4% of the Dutch population suffer from psoriasis. Psoriasis is a chronic recurrent skin disorder characterised by erythematosquamous lesions (plaques). Usually the affected areas are few, but occasionaly the disease is more generalised. Psoriasis can be treated topically by application of creams and ointments, for instance corticosteroids and vitamin D_3_. For most patients topical therapy will suffice. However, for some patients the area involved is so extensive that local treatment is not feasible. In other cases the skin lesions no longer respond to topical treatment. In that case the dermatologist may start irradiation with ultraviolet (UV) light or prescribe systemic medication. UV irradiation of the skin can be performed with different types of UV light: e.g. UVA, broadband UVB, or narrowband UVB. UVB irradiation is usually prescribed as a single therapy. However, adjuvant use of topical therapy may be continued.

UVB therapy has considerable consequences for the patient because it is nearly always carried out in a hospital outpatient clinic. The UVB irradiation itself normally takes only a few minutes, but to receive the irradiation patients have to travel to the outpatient department during working hours two to three times a week. In general it is a relatively time-consuming treatment. For hospital personnel as well, this mode of therapy demands a considerable investment of time. They have to determine the dosage, set the machine to the proper dosage, and fill out the medical records for each patient visit.

To overcome the drawbacks of UVB treatment in the outpatient clinic, home UVB phototherapy was introduced over 25 years ago [1-4]. Ever since, however, the safety and effectiveness of home UVB have been the subject of debate [5-13]. Despite all discussion, increasing numbers of dermatologists seem to be prescribing home UVB phototherapy to their patients.

In an earlier study we listed all known publications and studies on home UVB phototherapy, and conducted a survey among dermatologists [14]. We found that in the Netherlands home UVB is currently prescribed to approximately 5% of the UVB-treated patients, with some dermatologists prescribing it in 100% of the cases. We also demonstrated that there is no firm evidence that would either support or dissuade from prescribing home UVB phototherapy. Glaringly absent is any randomised research in this area. This lack of research has resulted in ongoing discussions and the dissemination of personal, non-evidence based opinions, especially with regard to issues like effectiveness, side effects and cost [14]. Thus, in general, home UVB phototherapy remains a debated treatment. We concluded that only randomised research on home UVB phototherapy as compared to UVB treatment in an outpatient setting could resolve the issue [14].

In this paper, we describe a randomised pragmatic trial that we are currently conducting, a national trial on home UVB phototherapy for psoriasis. The Dutch acronym for this trial is PLUTO (Psoriasis: Landelijk UVB Thuisbelichtings Onderzoek). The trial is designed to evaluate the impact of home UVB phototherapy versus conventional outpatient UVB phototherapy on effectiveness, quality of life and cost-effectiveness. The focus is on narrowband UVB treatment (TL-01 lamps). The study tests the hypothesis that home UVB phototherapy is as effective as outpatient UVB phototherapy. We further expect a better quality of life when patients are treated at home, and we hypothesize that home treatment will have similar or reduced total costs. This article presents the design of this trial.

## Methods

### Objective

The aim of this study is to compare home UVB (TL-01) phototherapy with the current outpatient UVB (TL-01) phototherapy for patients with psoriasis. This objective was specified by the following research questions. Compared to outpatient UVB phototherapy:

1. Is home UVB phototherapy for patients with psoriasis equally effective?

2. Does home UVB phototherapy yield a better quality of life?

3. Are costs for home UVB phototherapy higher, lower or similar?

4. Is home UVB phototherapy cost-effective?

### Design

We conducted a pragmatic randomised parallel group single-blind multi-centre trial among psoriasis patients eligible for narrowband UVB (TL-01) phototherapy. Patients were randomly allocated to two groups, thus obtaining two treatment groups of equivalent prognosis. One group was given home UVB phototherapy and the other UVB phototherapy in the outpatient department of the participating hospitals.

The design was chosen to be 'pragmatic' in order to compare the two treatments under the conditions in which they would be applied in daily practice [15]. Accordingly, in our pragmatic trial we randomised the patients into two treatment groups, but we did not impose a prespecified treatment regimen on the participants. Instead, we urged dermatologists to carry out the assigned UVB treatments as they would normally, and thus to act in accordance with their own views. Consequently, all of the implicit differences in the two treatments were compared, including factors such as frequency of irradiations, dosage, compliance, concomitant medication, support and equipment. We did not control for these and other possible differences relating to the treatment, because they cannot be controlled for in a real life situation.

The locations of the two treatments (at home versus in the outpatient department) of course made blinding of the participants impossible. Because of the pragmatic design of the study it was not desirable to blind the dermatologist. However, we arranged for the extent and severity of the psoriasis to be assessed by an independant research nurse blinded to treatment form.

### Study population

Psoriasis patients who were clinically eligible for narrowband UVB (TL-01) phototherapy ànd who had had this therapy prescribed by their own dermatologist were invited to participate in the study. All inclusion and exclusion criteria are displayed in table 1.

**Table 1:** Eligibility criteria. Study participants were subject to the following inclusion and exclusion criteria.

**Inclusion criteria**:
1. Guttate or plaque psoriasis, clinically eligible for narrowband UVB (TL-01) phototherapy;
2. Willing to undergo treatment according to randomisation.

**Exclusion criteria**:

1. No informed consent:
• age below 18 years;
• not willing to accept one of the two treatments offered;
2. Practical reasons:
• not able to receive one of the two treatments offered (e.g. lack of space at home/living too far from hospital etc.);
• analphabetism (unable to read the patient information and the questionnaires, unable to provide written answers and written informed consent);
• lack of command of the Dutch language;
• not in possession of a telephone.
3. Expected non-compliance:
lack of understanding of what the study/treatment is about, and its potential consequences.
4. Medical contraindications:
• Malignancy of the skin in the past/at present;
• known UVB-allergy or chronic polymorphic photodermatosis;
• use (at time of inclusion) of medication with known phototoxic or photoallergic properties;
• use (at time of inclusion) of systemic antipsoriatic medication (cyclosporin, methotrexate, neotigason, fumaric acid);
• history of exposure to ionising radiation.

The main selection criterion of being "clinically eligible for narrowband UVB treatment" was purely pragmatic and was left to the discretion of the participating dermatologist. However, dermatologists were explicitly discouraged from increasing the number of their prescriptions on behalf of the study. No financial benefit or other compensation was offered to participating dermatologists for their efforts; this to avoid any conflict of interest and to include only those patients who would otherwise have received narrowband UVB treatment. Likewise, the patients also received no material compensation for their participation in the study.

### Sample size

The sample size could not be calculated on the basis of presumed differences in effectiveness as there is no clear data available on the possible differences in treatment effects. However, based on the data available from a 1993 pilot study [16] and recent experience with home UVB phototherapy, we in fact expected the effectiveness of both therapies to be similar. The sample size was therefore calculated in accordance with a negative trial approach [17]. We considered a 50% or greater improvement in the psoriasis severity from baseline to be a relevant clinical response. A systematic review by Spuls et al. indicates that approximately 85% of the patients treated with UVB show at least a 50% improvement in their psoriasis [18]. Thus, with N = 90 per per treatment group and at α = 0.05 and β = 0.20 (power 80%), we would be able to show a difference in effectiveness of 15% or more; i.e., a reduction in effectiveness from 85% to 70% or less should be distinguishable. To allow for missing data and losses to follow-up (i.e. withdrawals, incomplete case register forms) we aimed at 100 patients per group, 200 in total. To obtain accurate estimates of the cumulative costs of UVB treatment a consecutive sample of 100 patients (50 per group) was considered to be sufficient, because little variation was expected in treatment duration, number of UVB irradiations, and use of concomitant medication.

### Recruitment

#### Hospitals

We planned to include two hundred patients in approximately 2 years' time, starting October 2002. To achieve this, several hospitals were invited to join the study. Initially five university hospitals and one closely related non-university hospital agreed to participate. Later on, when inclusion of patients fell short of expectations, another eight hospitals were recruited from the same districts or nearby. In total 14 hospitals took part in the trial; 5 university hospitals and 9 non-university hospitals.

#### Patients

When the dermatologists of the collaborating hospitals prescribed UVB phototherapy to a patient, they checked eligibility for the trial using the above-mentioned list of inclusion and exclusion criteria (table 1). If all criteria were met, patients were informed about the possibility of participating. If the patient was interested, he/she received written information to take home. The same day the central coordination centre (UMC Utrecht) was provided with the name and phone number of the patient.

After 1–2 days the investigators at the central coordination centre contacted the patient by telephone and provided additional information. During this conversation the principle of randomisation (no choice of treatment) was explained at length, and eligibility criteria were checked again. If after reading and hearing the information and being allowed to ask additional questions patients were still interested in participating, a visit for inclusion and informed consent was scheduled as soon as possible. Patients not included in the trial were allowed to start their UVB therapy of choice without any further delay.

### Randomisation procedure

Every patient eligible for UVB phototherapy who was willing to participate in the study was registered at the central coordination centre. After providing informed consent and registration of baseline data a randomisation number corresponding with either home or outpatient phototherapy was drawn from a computer-generated list. Randomisation took place using stratified randomisation, in particular the minimisation method described by Pocock [19]. This method assigned the two treatments taking into account the recruiting hospital and possible previous experiences with UV phototherapy. After randomisation, both patient and dermatologist were informed of the assigned treatment, and this treatment was started according to standard procedures.

### UVB therapy and equipment

#### In the outpatient department

Patients randomised to the group treated in the outpatient department received the UVB treatment in their own hospital. The type of irradiation was restricted to narrowband UVB (TL-01 tubes) [20]. All hospitals used their own treatment schedules and their own (full circle) cabins. Some types of cabins had UV indicators measuring irradiation intensity (mW/cm^2^); others did not and measured only treatment time. For cabins with intensity indicators, treatment schedules were formulated in dosage (J/cm^2^). For cabins without intensity indicators, treatments were prescribed in units of time (seconds). Neither equipment nor schedules were modified for the trial. The frequency of irradiation was 2–3 times a week, depending on the hospital.

#### At home

When patients were assigned to have home UVB phototherapy, the investigator placed an order with one of the two home care organisations that provide the vast majority of home UV phototherapy in the Netherlands [14]. Orders were divided equally between the two organisations, taking into account equal distributions per hospital and the preferences of the patient's insurance company (reimbursement). The UVB treatment was administered in the patient's home, using equipment provided by one of the home care organisations. The home phototherapy units used were Waldmann UV-100 units with TL-01 lamps. This device comprises a semi-circular arrangement of lamps. These units do not have an irradiation intensity indicator; therefore treatments were prescribed in units of time. The patients were instructed and supervised in the use of the equipment by the nursing staff of the home care institutions. The treatment schedules were the schedules normally used by those institutions. Neither equipment nor schedules were modified for the trial. The frequency of irradiation was at least 3–4 times a week (i.e. once every 2 days, sometimes starting daily).

#### In general

In all cases the initial treatment plan was narrowband UVB phototherapy according to randomisation. No prespecified treatment regimen was imposed on the participants and adjuvant use of topical therapy was allowed to continue throughout the trial.

No other additional treatments or changes to the original treatment plan were intended. However, in order to compare the two UVB treatments under practical conditions and to reflect clinical reality, alterations to the initial treatment plan were allowed if the dermatologist decided they were necessary. As such, all treatment changes originating after inclusion and randomisation were permitted. For instance, starting any type of medication after inclusion, even systemic medication, was not considered a reason for exclusion if the dermatologist considered this treatment change necessary. Also, temporarily starting outpatient phototherapy while waiting for placement of a home phototherapy unit was allowed.

### Outcome assessment

To answer the separate research questions, several outcome assessments had to be performed during the trial. The majority of the outcomes were measured using questionnaires or were assessed by an independent, blinded research nurse.

#### Effectiveness

We assessed the effectiveness of both treatments using the Psoriasis Area and Severity Index (PASI) [21] and the Self-Administered PASI (SAPASI) [22]. Both indices evaluate the severity of the psoriatic lesions ànd the area of psoriatic involvement. Their scores vary from 0.0 (no lesions at all) to 72.0 (complete erythroderma of the severest possible degree) [21]. An independent and blinded research nurse administered the PASI during several patient visits to the outpatient clinic. The SAPASI was easier to collect using a questionnaire and was used as an equivalent of the PASI as well as an indicator of the patient's own impression of the extent of the psoriasis lesions [22,23] Both the PASI and the SAPASI were assessed for the whole body, including the lower legs and the scalp. We also determined skin type according to Fitzpatrick's classification of skin phototypes [24,25] and collected data on concomitant use of medication, side effects, demographics and past medical history.

#### Dosimetry

We routinely measured the light intensity (*J/cm^2^*) of all UVB equipment from the hospitals with a small portable Waldmann UV meter, type 585 100 (Villingen, Schwenningen, Germany), referred to as meter A. If the cabin had an irradiation intensity indicator, we compared its reading with our own measurements. To collect information about calculated treatment doses (*mW/cm^2^*), we made copies of the treatment charts of the participants treated in the outpatient departments.

The home care organisations measured the light intensity of every unit before the first irradiation and after the last irradiation, using their own small Waldmann UV meters, all type 585 100, referred to as meters B. At the end of the trial we collected these measurements and compared their meters (B) with our own Waldmann UV-meter (A), calibrated with the High Accuracy UV-Visible Spectroradiometer, type OL 752 (Orlando, Florida, U.S.A.).

Both groups of participants kept a record of their treatment times in their diary. For all patients we calculated standardised cumulative doses using the intensity measurements together with the individual treatment charts and/or diaries. The way cumulative doses were standardised in this trial is described in table 2.

**Table 2:** Calculation of standardised cumulative doses for the three different situations in the trial.

**Situation**	**Treatment location**	**UVB equipment**	**Treatment schedule**	**Cumulative Dose (CD)**	**Standardisation factor F**
1	In the hospital	With intensity indicator	in J/cm^2^	*CD *= *CD*_*h *_× *F*	F=IAIh×IsrIcA MathType@MTEF@5@5@+=feaafiart1ev1aaatCvAUfKttLearuWrP9MDH5MBPbIqV92AaeXatLxBI9gBaebbnrfifHhDYfgasaacH8akY=wiFfYdH8Gipec8Eeeu0xXdbba9frFj0=OqFfea0dXdd9vqai=hGuQ8kuc9pgc9s8qqaq=dirpe0xb9q8qiLsFr0=vr0=vr0dc8meaabaqaciaacaGaaeqabaqabeGadaaakeaacqWGgbGrcqGH9aqpdaWcaaqaaiabdMeajnaaBaaaleaacqWGbbqqaeqaaaGcbaGaemysaK0aaSbaaSqaaiabdIgaObqabaaaaOGaey41aq7aaSaaaeaacqWGjbqsdaWgaaWcbaGaem4CamNaemOCaihabeaaaOqaaiabdMeajnaaBaaaleaacqWGJbWycqWGbbqqaeqaaaaaaaa@3DD2@
2	In the hospital	Without intensity indicator	in time (seconds)	*CD *= *CTT *× *I*_*A *_× *F*	F=IcAIcA×IsrIcA MathType@MTEF@5@5@+=feaafiart1ev1aaatCvAUfKttLearuWrP9MDH5MBPbIqV92AaeXatLxBI9gBaebbnrfifHhDYfgasaacH8akY=wiFfYdH8Gipec8Eeeu0xXdbba9frFj0=OqFfea0dXdd9vqai=hGuQ8kuc9pgc9s8qqaq=dirpe0xb9q8qiLsFr0=vr0=vr0dc8meaabaqaciaacaGaaeqabaqabeGadaaakeaacqWGgbGrcqGH9aqpdaWcaaqaaiabdMeajnaaBaaaleaacqWGJbWycqWGbbqqaeqaaaGcbaGaemysaK0aaSbaaSqaaiabdogaJjabdgeabbqabaaaaOGaey41aq7aaSaaaeaacqWGjbqsdaWgaaWcbaGaem4CamNaemOCaihabeaaaOqaaiabdMeajnaaBaaaleaacqWGJbWycqWGbbqqaeqaaaaaaaa@4022@
3	At home	Without intensity indicator	in time (seconds)	CD=CTT×IB1+IB22×F MathType@MTEF@5@5@+=feaafiart1ev1aaatCvAUfKttLearuWrP9MDH5MBPbIqV92AaeXatLxBI9gBaebbnrfifHhDYfgasaacH8akY=wiFfYdH8Gipec8Eeeu0xXdbba9frFj0=OqFfea0dXdd9vqai=hGuQ8kuc9pgc9s8qqaq=dirpe0xb9q8qiLsFr0=vr0=vr0dc8meaabaqaciaacaGaaeqabaqabeGadaaakeaacqWGdbWqcqWGebarcqGH9aqpcqWGdbWqcqWGubavcqWGubavcqGHxdaTdaWcaaqaaiabdMeajnaaBaaaleaacqWGcbGqcqaIXaqmaeqaaOGaey4kaSIaemysaK0aaSbaaSqaaiabdkeacjabikdaYaqabaaakeaacqaIYaGmaaGaey41aqRaemOrayeaaa@4108@	F=IcAIcB×IsrIcA MathType@MTEF@5@5@+=feaafiart1ev1aaatCvAUfKttLearuWrP9MDH5MBPbIqV92AaeXatLxBI9gBaebbnrfifHhDYfgasaacH8akY=wiFfYdH8Gipec8Eeeu0xXdbba9frFj0=OqFfea0dXdd9vqai=hGuQ8kuc9pgc9s8qqaq=dirpe0xb9q8qiLsFr0=vr0=vr0dc8meaabaqaciaacaGaaeqabaqabeGadaaakeaacqWGgbGrcqGH9aqpdaWcaaqaaiabdMeajnaaBaaaleaacqWGJbWycqWGbbqqaeqaaaGcbaGaemysaK0aaSbaaSqaaiabdogaJjabdkeacbqabaaaaOGaey41aq7aaSaaaeaacqWGjbqsdaWgaaWcbaGaem4CamNaemOCaihabeaaaOqaaiabdMeajnaaBaaaleaacqWGJbWycqWGbbqqaeqaaaaaaaa@4024@

*CD *= Cumulative Dose (*J/cm^2^*) standardised for the study.

*CD*_*h *_= Cumulative Dose (*J/cm^2^*) as calculated by the hospital (retrieved from treatment chart).

*CTT *= Cumulative Treatment Time (*s*) as retreived from the diary and/or treatment chart.

*F *= correction factor for standardisation.

Measurements on location:

*I*_*A *_= Intensity (*mW/cm^2^*) as measured by the investigators for each hospital UVB unit with portable intensity meter A.

*I*_*h *_= Intensity (*mW/cm^2^*) as simultaneously measured by the hospitals' UVB unit irradiation intensity indicator.

*I*_*B*1 _= Intensity (*mW/cm^2^*) measured by the home care organisations before start of first treatment, using their meters (B).

*I*_*B*2 _= Intensity (*mW/cm^2^*) measured by the home care organisations after finishing last treatment, using their meters (B).

Measurements during calibration:

*I*_*cA *_= Intensity (*mW/cm^2^*) as measured during calibration by the investigators' portable intensity meter A.

*I*_*cB *_= Intensity (*mW/cm^2^*) as measured during calibration by the intensity meters (B) of the home care organisations.

*I*_*sr *_= Intensity (*mW/cm^2^*) as measured during calibration with the High Accuracy Spectroradiometer type OL 752

#### Quality of life (QoL)

To assess quality of life we used one disease-specific questionnaire and several universal questionnaires. The standard questionnaires that were used were:

• Psoriasis Disability Index (PDI) [26,27]

• Short Form-36 (SF-36) [28,29], and

• EuroQol (EQ-5D) [30,31]

The PDI is a short questionnaire consisting of 15 questions regarding disability due to psoriasis. Answers are recorded on a seven point linear scale where '1' indicates no disability and '7' indicates maximum disability. Therefore the maximum potential PDI score is 105, with a minimum of 15 [27]. One of the generic QoL questionnaires used in the study is the SF-36, a 36-item questionnaire yielding a profile of 8 dimensions. All dimensions range in score from 0 to 100, with higher scores indicating a higher level of health status. By adding weighted combinations, the 8 scales can be combined into a physical and a mental component summary score. The EQ-5D is also a generic quality of life questionnaire. It was developed to assess the impact of a disease in terms of multi-attribute value judgements yielding one overall value judgement, a so-called utility score ranging between 0 (dead) and 1 (optimal health) [30,31]. When utility scores are plotted against time, the Area Under the Curve (AUC) reflects the Quality Adjusted Life time usually expressed in Years (QALY's). Thus QALY's will be calculated as follows: utility scores from two timepoints will be linearly interpolated (i.e. summed and devided by two). This outcome will be multiplied by the time difference. This procedure will be repeated for all parts of the curve, and the outcomes of all parts of the curve will be summed and ultimately yield an estimate of the entire AUC. In this way QALY's will be calculated for all patients, and a mean and standard error of the mean (SEM) will be determined.

Besides these standard questionnaires, we designed a brief "Burden of treatment" (BOT) questionnaire with 4 questions on the perceived burden of the UVB treatment (especially the specific burden of the treatment method and the burden of the time lost to treatment), and we developed a questionnaire on patient satisfaction. The patient satisfaction questionnaire asked about:

• waiting times,

• perceived improvement of the psoriasis lesions,

• satisfaction with the treatment as a whole,

• satisfaction with the final treatment result,

• satisfaction with the rate of improvement,

• satisfaction with the nurses' supervision,

• perceived extent of side effects,

• perceived safety of the treatment,

• perceived advantages and drawbacks of both modes of therapy, and

• preferences with regard to future UVB treatment, if necessary (home UVB phototherapy versus UVB treatment in the hospital).

#### Costs

Estimation of costs will be based partially on the actual cost of the resources used, for instance the rental price of home UVB light panels, the cost of using hospital equipment, and travel expenses among others. Some indirect costs-such as the loss of work time due to decreased efficiency or absenteeism-were assessed using the "Health and Labour Questionnaire" [32], a general introductory questionnaire and the previously mentioned diary. These two questionnaires will supply information on time lost or expenses incurred for the treatment of the psoriasis, for instance the cost of transportation to a dermatologist or general practitioner. The diary on the other hand provides information on the frequency of these types of expenses during the treatment period. From the patients' pharmacists we obtained data on the use of medications and their prices. The friction cost method [33] will be applied to assess the losses to society due to sick leave.

#### Cost-effectiveness

A direct comparison will be made between the effectiveness of the two therapeutic modalities and their associated cost. Incremental cost per additional patient treated successfully and costs per QALY gained will be estimated. Cost-effectiveness (CE) will be calculated for a time horizon of 12 (11–13) months after randomisation/inclusion. The quality of life (QoL) questionnaires were no longer administered after cessation of irradiation. Based on the association between clinical symptoms and QoL, the impact in terms of QoL for the remaining follow-up, i.e. up to 12 months, will be extrapolated using regression models. Subsequently, cost per QALY can be calculated. In case the difference in effectiveness is less than 15%, we will consider this as equal effectiveness and will limit the economic evaluation to a cost minimisation analysis.

### Measurement planning

**Figure 1:**
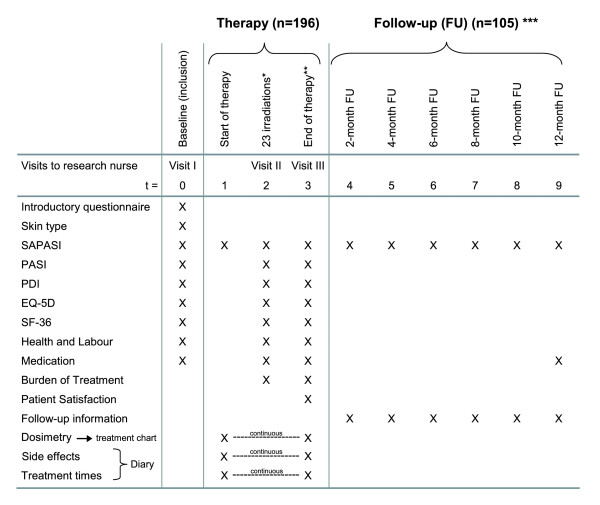
**Timetable**. Schematic representation of successive time points for data collection, reported for all outcome measures and questionnaires. * 23 irradiations: outcome measurement was planned at approximately 23 irradiations, with a minimum of 20 and a maximum of 26 irradiations. ** End of therapy: measurement was planned at the end of the treatment. When more than 46 irradiations were needed, measurement was planned at 46 irradiations. *** Follow-up: Starting at the end of the therapy (or at the 46^th ^irradiation, see **), follow-up measurements were planned every 2 months, for up to 1 year after the last irradiation.

Outcome measurement was planned according to time points specified in the timetable in figure 1. Briefly, the procedure was as follows: When patients were willing to participate, we arranged a visit with an independent research nurse. During this visit, patients signed a consent form, baseline data were recorded and instructions were given on the use of the diary and questionnaires.

Immediately after this first visit, patients were randomised using the baseline data (information on the recruiting hospital and any previous experience of the patient with UV therapy were used to determine the randomisation strata). Both patient and attending dermatologist were informed about the type of treatment assigned, and the treatment was started accordingly. When (sometimes after a waiting period) the therapy was started, the patients did a SAPASI-assessment and began using the study diary. At approximately 23 (20–26) UVB treatments ànd at the end of the therapy (or 46 treatments) the patients received a series of questionnaires at home. Visits to the previously mentioned research nurse were scheduled for the same time points. Throughout the whole trial, the independent research nurse was not informed about the randomisation results and therefore remained blinded.

For all UVB therapies lasting longer than 46 treatments, a cutoff point of maximally 46 treatments was used to establish effectiveness. The choice of 46 as the maximum number of treatments was derived from unpublished data from the home care organisations about the treatment duration of their patients.

At the end of the therapy (or 46 treatments), 105 patients were followed for 12 more months to monitor long term effectiveness and cost. The patients received a short questionnaire at home every 2 months and returned this questionnaire by mail. At the end of the follow-up, information on medication use during the trial period was retrieved retrospectively for each patient from his/her pharmacist.

### Ethics & informed consent

The final protocol was approved by the Medical Ethics Committees of all participating hospitals. Patients were able to quit the study at any time. Informed consent was obtained after the study objectives, types of therapy, benefits and risks, and the concept of randomisation had been explained extensively. The study was performed according to the principles of Good Clinical Practice [34].

### Statistical analysis

All data will be analysed according to the intention to treat principle, meaning all included patients will be analysed according to the group they were randomised to. This includes patients who dropped out or changed therapy. The statistical methods that will be used are chosen in accordance with the type of data that is available. For continuous data differences and their 95% Confidence Intervals (95% CIs) will be presented. In case of binary outcomes, differences in proportion with 95% CIs will be calculated. Whether randomisation was successful will be determined by assessing comparability of baseline characteristics. No formal statistical tests are foreseen. In the event apparent differences are noted, a multivariate analysis will be performed to adjust for potential confounding.

#### Effectiveness

Assessment of effectiveness of both UVB treatments will take place through calculation of the PASI and SAPASI-scores for both treatment modalities at regular points in time, and subsequently calculating differences in (SA)PASI scores over time. Possible differences in effectiveness between the two therapies will be established through comparison of differences in (SA)PASI scores inclusive of 95% CIs. The side effects of the therapy were reported for each irradiation as being present ("yes") or not being present ("no"). For each type of side effect the answers will be compared on a group level and presented with their 95% CIs.

#### Dosimetry

Cumulative dosimetry will be determined as formulated above in table 2. Comparison of cumulative doses between the treatment groups will be done by calculating the difference in mean dose with its 95% CI.

#### Quality of life (QoL)

Continuous sum-scores of the QoL questionnaires will be compared by calculating differences in QoL and their 95% CIs. Changes in QoL will also be compared across groups.

#### Costs

Both costs as reimbursed by the health insurance companies and actual costs as calculated by including all expenses (direct and indirect) will be compared for the two treatments as difference in cost with a 95% CI. The uncertainty associated with the point estimates will be evaluated using bootstrapping [35].

#### Cost-effectiveness

Initially the trial estimate of the incremental cost per additional patient with successful outcome will be assessed. Similarly, the cost per QALY gained will be estimated. To assess uncertainty with regard to incremental cost-effectiveness a standard bootstrap technique will be applied [35]. The trial data will be randomly sampled with replacement from the original dataset 1000 times. For each bootstrap sample the incremental costs and effects will be calculated and plotted (costs on the y-axis and effects on the x-axis). Thus an integrated presentation of the mutually dependent cost and effect differences is obtained that may be interpreted as a direct reflection of the uncertainty, i.e. a two dimensial dispersion, with regard to the incremental cost-effectiveness ratio. Using this so-called CEA Plane (Cost-effectiveness Analysis Plane), an inference regarding the likelihood of one treatment being more cost-effective than the other can be made [36].

#### Other data

These include concomitant use of medication, data on patient satisfaction and on the burden of both treatments. These data will also be compared across groups and be presented as differences with 95% CIs.

## Results

Recruitment of patients was stopped after 2 years and 2 1/2 months. Initially 252 possible participants were recruited by the participating hospitals. Of these 252 patients, 56 were excluded from starting the trial for a variety of reasons. Thirty-three (33) patients had a clear preference for either home or hospital-based therapy and refused to submit to being randomised. Another 11 patients did not want to participate in a study, 6 patients did not fulfill the inclusion criteria or met one of the exclusion criteria, 2 had already started UVB therapy, 2 persons decided not to be treated with UVB, and 2 patients were excluded for other practical reasons.

Thus, in total 196 patients (252–56) were included in the study. A consecutive sample of 105 patients was followed for 12 months after the end of the treatment in order to gain sufficient data about the costs incurred after the treatment. Randomisation according to the minimisation method [19] was successful. The two factors accounted for, their levels and the number of assigned therapies are shown in table 3.

**Table 3:** Results of the randomisation procedure.

		**Number of patients**		
**Factor**	**Level**	**Hospital**	**Home**	**Total**
**Hospital**	UMC Utrecht	24	25	49
	Hilversum Hospital	14	15	29
	Academic Hospital Maastricht	9	9	18
	Diakonessen Hospital Utrecht	9	8	17
	Meander Hospital Amersfoort	7	8	15
	Groene Hart Hospital Gouda	6	5	11
	AMC Amsterdam	5	5	10
	Erasmus MC Rotterdam	5	4	9
	VUmc Amsterdam	4	5	9
	Gelre Hospital Apeldoorn	5	3	8
	Diakonessen Hospital Zeist	3	4	7
	Reinier de Graaf Hospital Delft/Voorburg	4	3	7
	AntoniusMesosGroup Hospitals Nieuwegein/Utrecht	2	2	4
	Lucas Andreas Hospital Amsterdam	1	2	3

**Previous UV phototherapy**	Yes	50	50	100
	No	48	48	96

Stratified randomisation, in particular the minimisation method described by Pocock [19] was used, which took into account (1) the recruiting hospital, and (2) possible previous experience of the patient with UV therapy. This table shows the results of the treatment assignment by the two factors for 196 patients.

The therapy took place according to randomisation for 184 patients. Five (5) patients switched therapy (protocol violators), of which four patients switched to home UVB phototherapy. Another seven (7) patients never started therapy. Of those seven patients, four (4) had their lesions improve during the waiting period before treatment could be started and did not need UVB treatment after all (three of them were randomised to the group receiving home UVB treatment). The other three (3) that did not receive therapy withdrew respectively because of agoraphobia, not wanting to participate in the study, and not wanting any treatment during pregnancy. Two of them were assigned to have outpatient phototherapy.

## Discussion

This article presents the design of the first randomised controlled trial of home UVB phototherapy for psoriasis. The design of this trial was 'pragmatic', which means that we did not adapt daily practice to conform to a specific protocol: the two treatments were compared under the conditions in which they would be applied in daily practice [15]. In contrast to a pragmatic trial, an 'explanatory' trial studies treatments under controlled idealised or equalised conditions, preferably by means of a double-blind placebo-controlled study design and a rigid research protocol [15]. Thus, explanatory trials aim at providing information on the effects of a single difference in treatment, while a pragmatic trial compares two treatment strategies as a whole [15]. Consequently, in a pragmatic trial the treatments will be carried out just as they would have been without the trial, and therefore the advantage of a pragmatic design is the assurance that the results can easily be generalised to the target population. As a result, this trial will make a valuable and important contribution to the evidence base for the use of home UVB phototherapy for patients with psoriasis. It will produce a solid estimate of the effectiveness of home UVB treatment compared to UVB phototherapy in an outpatient setting.

Of the five previously conducted studies on home UVB treatment, only two compare two groups of patients [14]. Our study addresses two weaknesses encountered in those earlier studies. First of all, our trial has a parallel group design in order to compare the two treatments during the same seasons of the year. Secondly, to obtain two similar groups of patients and thus prevent selection bias, assignment of treatments is by randomisation. As such, this study is the first to perform a parallel group randomised comparison of home UVB phototherapy with UVB treatment in an outpatient setting.

Another strength of the study is that it is designed to compare the two treatments as they are carried out in daily practise (pragmatic design), guaranteeing a good generalisability of the results. A further substantial difference with other trials in this field is that in addition to the effectiveness of the two therapies, the impact of each treatment on quality of life and on its associated costs is assessed. A cost-effectiveness analysis will be conducted to investigate the aspects of expense and effectiveness together. The trial will also provide information about patient satisfaction, travel time, side effects, cumulative dosimetry, concomitant use of medication, waiting periods and total treatment duration. The manner in which data collection was planned throughout the trial ensures that both groups can be compared during the treatment period without important differences in the number of irradiations. During the follow-up the measurements are all comparable with regard to the time interval since the last irradiation.

There are however also some weak points which bear discussion. For instance, because the measurement planning does not use fixed time points starting from inclusion, for most patients the outcome at 12 months after inclusion (used to calculate cost-effectiveness) will have to be interpolated from the two adjacent measurements. These two adjacent measurements are only two months apart, thus providing a solid basis for interpolation. Also, the EQ-5D questionnaire was only administered until the end of the treatment and not during the remaining follow-up period, making direct calculation of QALY's for this period impossible. We will have to rely on extrapolation from the treatment period. Another issue for discussion is that during the trial it was impossible to keep a record of all patients with psoriasis who were prescribed TL-01 UVB treatment but who were nòt referred to the central coordination centre. We therefore do not know the reasons for non-referral.

Overall, we feel that this trial has many benefits and will prove to be a very valuable addition to the current literature in the field. It is the first trial to compare these two commonly used treatments directly in a randomised parallel group design. Moreover, the study design allows for more than one objective. Besides clinical effectiveness, it also evaluates quality of life and cost-effectiveness. Being a pragmatic trial, it will be easy to generalise the results of this study to the target population. With 196 participants, the study is adequately powered to detect a 15% reduction in effectiveness, and the other outcome parameters can also be determined adequately. The results of this study will be available in 2006.

## Competing interests

The author(s) declare that they have no competing interests.

## Authors' contributions

PS, VS, HvW, EB and CBK were responsible for the research question. PS and EB drafted the initial study protocol. MK, PS, VS, HvW, EB and CBK participated in the conceptualisation and implementation of the trial. MK and VS coordinated the whole trial. MK was responsible for writing this manuscript. All authors read, commented on and approved the final manuscript. VS is the guarantor of this paper.

## Pre-publication history

The pre-publication history for this paper can be accessed here:


